# Animal models of compulsion alcohol drinking: Why we love quinine-resistant intake and what we learned from it

**DOI:** 10.3389/fpsyt.2023.1116901

**Published:** 2023-03-24

**Authors:** Thatiane De Oliveira Sergio, Raizel M. Frasier, Frederic W. Hopf

**Affiliations:** Department of Psychiatry, Indiana University School of Medicine, Indianapolis, IN, United States

**Keywords:** compulsion alcohol drinking, α adrenergic receptors, β adrenergic receptors, quinine adulteration, footshock sensitivity

## Abstract

Alcohol Use Disorder (AUD) ranks among the most prevalent mental disorders, extracting ~$250 billion/year in the US alone and producing myriad medical and social harms. Also, the number of deaths related to problem drinking has been increasing dramatically. Compulsive alcohol drinking, characterized by intake that persists despite negative consequences, can be particularly important and a major obstacle to treatment. With the number of people suffering from AUD increasing during the past years, there is a critical need to understand the neurobiology related to compulsive drives for alcohol, as well as the development of novel AUD pharmacological therapies. Here we discuss rodent compulsion-like alcohol drinking (CLAD) models, focusing on the two most widely used adverse stimuli to model rodent compulsion-like responding, quinine adulteration of alcohol and footshook-resistant alcohol intake. For both cases, the goal is to uncover behavior patterns and brain circuits that underlie drive for alcohol even in the face of negative consequences. We discuss caveats, benefits, and potential brain mechanisms, of models for consequence-resistant responding for alcohol more generally, and especially highlight some advantages of quinine-resistance over footshook-resistance. Further, since this review contributes to a Special issue focused on Molecular Aspects of Compulsive Drug Use, we discuss our new findings showing how the noradrenergic system is related to CLAD responding. In particular, we comment on the importance of α1 and β adrenergic receptors (ARs) as potential targets for treating AUD.

## 1. Introduction

Alcohol consumption occurs widely in numerous cultural and social events, and, although it provides pleasure to many, a subset of people move from recreational use to development of drinking problems (1–4). Indeed, misuse of alcohol relates to many negative personal and social outcomes, which overall occur in high frequency (e.g., vehicle crashes, falls, sexually transmitted diseases, unintended or poor pregnancy outcomes, high blood pressure, cholesterol, heart and liver diseases), and costs a substantial amount of money worldwide from health care expenditures, lost work productivity, criminal justice, etc., (1, 5–11). In the United States alone, excessive alcohol use in 2010–2011 was responsible for ~95,000 deaths and ~$249 billion, or $2.05 per drink, and binge drinking accounted for 77% of these costs, or $191 billion (1). Also, in the European Union, alcohol ranks as the most harmful abused drug, followed by heroin and crack (12). Indeed, because alcohol is legal, it continues to extract outsized costs relative to other intoxicants.

More recent findings remain discouraging. A recent study found that excessive alcohol drinking is now the leading cause of death in young to middle-aged U.S. adults, accounting for 1 in 5 deaths among adults aged 20 to 49 years, and 1 in 8 deaths among adults aged 20 to 64 years (13). Also, these alarming findings can still be conservative, given that they are based on deaths related to alcohol conditions that were more clearly identified. It is also important to point out that this study examined excessive drinking levels before the COVID-19 pandemic, which itself has increased problem drinking as much as 30% (14–16). However, one cause for some hope is that alcohol intake in high school students has steadily dropped since the year 2000 or so^1^. Despite the alarming scale of alcohol drinking problems, only a few pharmacological compounds are approved for treatment (17, 18), and much remains unclear about the complexity of the neural and molecular mechanisms related to this disease.

We are particularly interested in compulsion-like responding for alcohol, where intake persists despite negative consequences, since this can be a major obstacle to treatment and a strong driver of excessive intake (3, 19–27). Also, problem drinking in women has risen dramatically in recent years (2, 28–30), and women often suffer greater consequences from their alcohol use (31–33). Furthermore, negative affect and anxiety-related conditions can strongly contribute to alcohol drinking (4, 34–38), at least in some individuals (39–42), and women have nearly twice the risk of developing an affect disorder (43–48) and have higher comorbidity of AUD and affect disorders (32, 35). Thus, to help develop better, personalized therapies (40), it is essential to understand sex differences and similarities in mechanisms underlying excessive drinking and its comorbidities (37, 38, 49).

It is also important to note that compulsion-like motivations are just one of several important drivers of problem alcohol drinking. For example, human studies have long examined possible mechanistic differences in so-called reward vs. relief drinking (39–42). Reward drives include alcohol cue reactivity, sensation-seeking, and impulsivity, and can be assessed by self-reported subjective stimulation and intoxication by alcohol. Relief drinking is focused on intake to relieve or stave off negative emotional or physical feelings. Like many other mental health conditions, there are conditioned cues, in our case for alcohol, that can strongly and more automatically elicit approach toward such alcohol cues (50–53) [see also Weafer et al. (54)]. In addition, these are not the only factors that can drive alcohol-directed behavior, and it is likely that a given problem drinker can exhibit different motives for alcohol at different times, or some combination of drives. Nonetheless, more automatic and reflexive responding has been considered an important aspect of compulsion-like drinking (3, 27, 55–57). In addition, groups have emphasized the fundamental role of conflict for compulsive aspects of addiction (19, 22, 58) (discussed in Section 2), as well as high motivation, reflected in part by continuation of responding despite the cost of dealing with negative consequences (3, 27).

Other aspects of compulsion, including considerations and caveats, are discussed in several recent reviews (59–63), and we predominantly agree with the points they detail in our publications (64–70). These include (1) considerations of over-focus on compulsion, to the detriment of overlooking other factors that can contribute to addiction (59–61, 65, 71) (above), (2) the importance of considering compulsion for alcohol when adapting the NIMH RDoC framework for addiction (3, 72, 73), and (3) the importance of agency and knowledge of choosing during recovery from compulsion-like drinking pattern (19, 20, 74, 75).

Finally, in identifying and targeting brain mechanisms thought to drive pathological alcohol drinking, we are particularly interested in identifying whether there might be pharmacotherapies which would be effective against compulsion-like drinking, especially in individuals with higher daily intake levels. This is in alignment with overall shifts in recent decades that recognize the importance of individual differences and personalized medicine.

## 2. Quinine-resistant drinking: Some advantages over footshock-resistance?

While clinical studies are essential for understanding alcohol drives in humans, they have important limitations, especially in the ability to have mechanistic interventions. Thus, rodent work more generally affords a number of specific and valuable methods to uncover critical aspects of brain circuitry that mediate compulsive drives for alcohol. For example, our group has used projection-specific optogenetic inhibition, using laser light to inhibit projections from specific cortical cells during different aspects of behavior (detailed below), and *in vivo* recording of brain activity patterns during CLAD and alcohol-only drinking (AOD) (70). Combined with local pharmacological inhibition during drinking, these preclinical interventions can help unravel the molecular and neural mechanisms related to CLAD (and excessive drinking more generally). In addition, as we detail below, behavioral microstructure, combined with other methods, likely provides some important insights into the action strategies rats utilize during different kinds of alcohol drinking, and perhaps even subjective state (discussed below).

### 2.1. Footshock-resistance as a CLAD model

Maintaining intake in the face of shock (footshock-resistance) is sometimes considered more of a gold standard to model human compulsion, including since shock is well-defined in its ability to evoke fear and as an aversive stimulus (25, 27, 76). Thus, we examined the brain circuitry that might underlie footshock-resistance for alcohol.

It would be especially valuable if we found evidence that a common circuit is important for CLAD across different adverse conditions and even across species. We have argued for the central importance of the Anterior Insula Cortex (AIC) system, which detects and helps respond to high importance situations, for addiction-related states and behaviors [detailed in Centanni et al. (66)], along with other reviews relating AIC to alcohol behaviors (77–79). More generally, AIC is a key regulator of the Salience Network, including medial prefrontal cortex, nucleus accumbens, amygdala, and other areas, which together play a broad role in importance-directed planning and action (66, 80–82). Thus, our projection-specific optogenetic studies (83) focused on rat putative Salience Network areas, and, importantly, we found that inhibiting either (1) AIC-to-nucleus accumbens or (2) medial prefrontal-to-nucleus accumbens significantly reduced footshock-resistant operant responding for alcohol; critically, these pathways have no impact on operant responding for alcohol-only (see below).

Interestingly, Grodin et al. (84) set out to develop a shock-resistant responding for alcohol paradigm in heavy drinking humans, and found a similar AIC/medial prefrontal/striatal circuitry for compulsive action for alcohol. While artificial, a similar AIC system is activated by imagining higher-risk (vs. lower risk) drinking in problem-drinking humans (85). This human-rodent concurrence in underlying brain circuits related to consequence-resistant responding for alcohol has increased our confidence that the rodent CLAD models we and others use may tap into circuits relevant to human consequence-resistant responding for alcohol. We also note that other regions, such as central nucleus of the amygdala, locus coeruleus, and periaqueductal gray, are likely important for compulsion-like aspects of addiction (3, 86–90).

Before addressing quinine, we want to further address the findings described above that AIC projections regulate CLAD but not AOD in rats (83). Similar to our studies in rats, disrupting AIC in male mice decreases CLAD but not AOD (91), and rat studies link AIC to level of CLAD expression (92) (see also Section 4 for orexin and CLAD vs. AOD). Similar, convergent human and rodent evidence implicates NMDARs in more pathological aspects of alcohol drives [detailed in Hopf (26), Simmler et al. (89); Section 4]. We interpret these findings in relation to discussions from clinical groups (22, 58) who propose that compulsion during addiction reflects conflict between desired reward and desire to avoid adverse consequences. Importantly, in this view, it is the conflict that recruits the Salience Network to play a role during compulsive drives for intake (22, 58). This use of AIC signaling to resolve and overcome conflict is a central part of our model, where CLAD involves need for greater attention on acting to obtain the reward, in our case alcohol (detailed below).

### 2.2. Quinine-resistance as a CLAD model

For alcohol studies, researchers have long tried to address adversity-resistant drive for intake by putting the bitter-tasting adulterant, quinine, into the alcohol. Here, we address some evidence that footshock- and quinine-resistance may be mechanistically related, then discuss several advantages of the quinine-alcohol drinking model.

**Figure 1:**
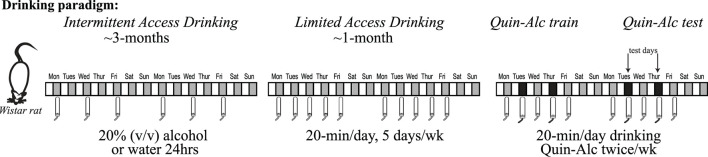
Intermittent two-bottle choice, access to alcohol, paradigm. Wistar rats have access to alcohol (20% v/v diluted in water) in the intermittent two-bottle choice paradigm. Briefly, three times a week (starting Monday, Wednesday and Friday around start of the dark cycle), rats have an 18–24 h period where alcohol is available concurrently with water. The alcohol and water bottle positions are alternated across days to prevent a position bias. Intermittent access continues for ~12 weeks, since longer-term intake is necessary to facilitate development of aversion-resistant alcohol intake. After ~3 months of intermittent access, rats are then shifted to Limited Daily Access two-bottle choice, with 20 min access to 20% alcohol or water Monday through Friday. After at least 2–3 wk limited daily access, rats have 2–3 alcohol-quinine sessions per quinine dose used to habituate to the novelty of quinine in alcohol. Experimental testing of quinine-resistant alcohol drinking then begins, typically with 2 test sessions per week.

Our studies described above show that AIC-to-nucleus accumbens and medial prefrontal-to-nucleus accumbens projections are critical for maintaining footshock-resistant alcohol intake, with no role in alcohol-only intake (83). Most importantly, in the same study, both neural projections also significantly promote quinine-resistant alcohol drinking. Thus, AIC-related salience circuits are critical for promoting both quinine-resistant and footshock-resistant alcohol drinking, suggesting a common mechanism, and validating the use of the simpler quinine-resistance model. In agreement, higher shock-resistance and quinine-resistance for alcohol are well correlated in rats (86, 93). Further, NMDA receptor modulators regulate quinine-resistant alcohol intake but not AOD in rats (at moderate doses), and parallel human studies implicate NMDA receptors in alcohol craving and drinking in treatment-seekers (ostensibly in conflict about drinking) but not in non-treatment-seekers [reviewed in Hopf (26) and Wegner et al. (94)]. Together, these results suggest that quinine-resistance in rat recruits a brain mechanism similar to footshock-resistance and perhaps also to human compulsion for alcohol. However, a recent study in genetically-selected, high-alcohol-drinking cHAP mice found much greater quinine resistance relative to shock resistance for alcohol (95). Nonetheless, the long-term, intermittent-access alcohol intake model we and others use in rat (Figure 1) exhibits several other features related to human AUD other than consequence-resistant responding for alcohol, including escalating intake (3, 96), sensitivity to compounds that reduce human drinking (96), withdrawal symptoms (although moderate) (97, 98), and front loading (indicating high motivation for alcohol) (67, 68, 99, 100). Thus, CLAD after long-term drinking in rats potentially reflects aspects of human problem drinking that are recapitulated in footshock-and quinine-resistant alcohol intake models.

One major goal of this commentary is to describe what we see as several advantages of quinine compared to footshock for CLAD studies. First, quinine-resistance is technically easier and faster since it does not require extensive daily operant training as in footshock-resistant alcohol drinking. Additionally, with much less personnel labor, quinine-resistance is also cheaper (the only thing necessary is a bottle with alcohol solution adulterated with quinine). We are further impressed with the robust ability to use quinine in alcohol to scale the level of aversion-resistance required to get alcohol. For example, the doses of quinine can be widely manipulated in the same individual rodent, and we frequently test multiple quinine doses per animal (across 10, 60, and 100 mg/L) in longer, randomized studies (67–70) (we consider 10 mg/L moderate-challenge and 60–100 mg/L higher-challenge). In our experience, it is more challenging to test different levels of shock and footshock-resistance in the same rat. In our previous study (83), half of male rats are resistant to alcohol when 1 in 8 responses is paired with shock, while nearly all rats strongly suppress consumption when the stimulus is delivered for 1 in 3 rewards. A similar “self-imposed abstinence” can be seen with ascending shock levels for cocaine (101, 102). We also attempted to do intermittent footshock synched to bottle licking for alcohol, and found our results quite variable (unpublished observations). One challenge with repeated testing of shock across conditions is possible sensitization after earlier shocks (27). However, even with these points, it could still be useful for some mechanistic studies of CLAD to test both quinine-resistance and shock-resistance.

One criticism about the quinine-adulteration model is that rodents could be less sensitive to bitter taste due the exposure of alcohol (59). However, several groups concur that male and female alcohol-drinking rats and mice greatly avoid consuming water adulterated with quinine levels that they will consume when in alcohol (65, 69, 103, 104), indicating robust aversion resistance to obtain alcohol.

### 2.3. Using quinine-resistant drinking to infer putative subjective states underlying CLAD

Our group has worked to assess different patterns within licking under different alcohol conditions that might give clues to underlying action strategies being used. Importantly, lickometry and microstructural analysis of different licking behaviors has long been used to attempt to provide valuable insights into what type of responding the rat is choosing to engage in. For example, one could argue that compulsion-like drinking reflects a state of higher motivation, since the individual is willing to tolerate cost and adversity to obtain the alcohol. Based on the classic lickometry literature [discussed in Darevsky et al. (67), Darevsky and Hopf (68), and detailed in a concurrently submitted review], we might predict longer and/or faster licking.

**Figure 2:**
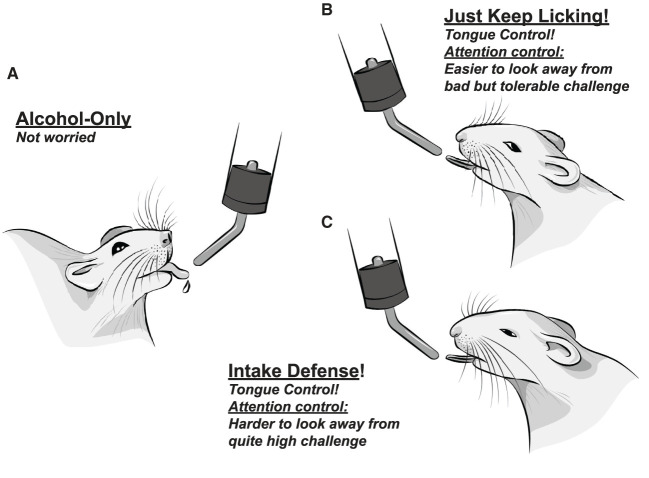
Somewhat anthropomorphized representations of proposed action, attention, and emotion regulation strategies utilized, including internal mental emotional states, during different rat drinking conditions. **(A)** Easy, alcohol-only drinking, with no overt adverse challenge. Several clinical groups propose that unchallenged intake recruits a more striatal circuit (22, 58), with less cortical role. **(B)** Drinking alcohol despite bad but tolerable challenge. Challenged rats show less variable responding in nearly every licking measure, including better control of tongue shape, relative to alcohol-only in (A). This is consistent with focusing on making more automatic, stereotyped actions. At the same time, we have proposed that the rat works to not put attention on the bad consequences (i.e., it successfully distracts itself); together, we call these the Head Down and Push model of responding despite challenge. **(C)** Drinking alcohol despite quite high challenge (enough to drop drinking by almost half). We propose that higher-challenge CLAD reflects Intake Defense: an idea that came from need to eat some food, even if it is spoiled [discussed in Darevsky et al. (67), Darevsky and Hopf (68) and Kaplan et al. (105)]. The goal is to just start, and push to get “enough” intake. Interestingly, high challenge drinking still shows better tongue control, and early initiation of drinking bouts. Since overall action plan, attentional regulation, and tongue control are all strongly regulated by the AIC, and lick measures can be observed under both high challenge **(C)** and less bad challenge **(B)**, we suggest that AIC's “commitment to act” is strongly maintained under high challenge; we propose that higher-challenge intake fails due to dis-coherence in the dorsal medial prefrontal cortex: see Darevsky et al. (67) and Darevsky and Hopf (68) and De Oliveira Sergio et al. (submitted) for further discussion.

In strong contrast with this prediction, we find that licking under aversive but tolerable (moderate) challenge is actually less variable in many lick measures (67, 68). While initially unexpected, more automatic and reflexive responding has been considered an important aspect of compulsion-like responding (3, 27, 55–57) (and identifying decreased variability in CLAD responding is one example of the strengths of microstructural analysis of licking). Moderate-challenge CLAD also shows significantly earlier bout start than AOD, and together we have proposed a Head Down and Push model of challenge-resistant responding. In particular, we propose that less variable responding reflects the putative goals of (1) focusing internal attention more on automatic action, and (2) ignoring negative consequences as best as possible, which would be helped by maintaining attentional control on performing the action; these are in contrast to the putatively cognitively simpler alcohol-only drinking (Figures 2A, B) (67, 68). Under higher-challenge alcohol drinking, total intake is greatly reduced (~40–50%), and lick timing becomes more variable and disrupted (the opposite of moderate-challenge). Interestingly, however, higher-challenge retains the better tongue control and earlier bout start seen with moderate-challenge (and which is different from alcohol-only) (Figure 2C), despite overall deficits in responding. Tongue control, attentional control, and overall action plans are likely strongly regulated by AIC (66–68). Thus, we consider these findings to indicate that AIC commitment to aversion-resistant alcohol intake remains strong under higher challenge [which was also observed in AIC firing patterns during CLAD; (70)]. In contrast, as we detail in another currently submitted review, we consider it likely that reduced intake and action organization under higher challenge is to due disruptions in medial prefrontal cortex activity [discussed in Darevsky et al. (67) and Darevsky and Hopf (68)].

We also would like to emphasize the purpose of our attempts to the infer both the action strategy utilized and also the subjective state of the rat under different drinking conditions. For example, along with the substantial AIC literature [reviewed in Centanni et al. (66)], we believe that the reduced variability in responding during moderate-challenge CLAD reflects a need for increased attentional control (focus on action to help decrease awareness of negative consequences). Such inferences about internal, subjective, and emotional states, if they are warranted by robust evidence, can be valuable in trying to understand the action (and likely emotion regulation) strategy that an individual is using under a given drinking condition. Importantly, we are in no way attempting to make light of AUD through our use of more colloquial language when attempting to describe the putative subjective state of the rodent. Our central goal is to understand the brain and psychological mechanisms of compulsion-like drives for alcohol (compared with alcohol-only), and using a more anthropomorphic description of particular inferred mental/emotional states is intended to be a pedagogical tool, however speculative, to help “get where the rat (or person) is coming from.” Indeed, others before us [e.g., Sanchis-Segura and Spanagel (106) and Meinhardt and Sommer (107)] have already made cartoon versions of different excessive alcohol drinking states, which were widely distributed, for use in both scientific and public talks. Their goal, as is ours, is both scientific (understanding the specific psychological drives that promote alcohol drinking) and educational, helping other scientists and even the lay public better understand addiction mechanisms, with the goal of increasing therapy development and implementation.

### 2.4. Using quinine-resistant drinking to assess development of CLAD

As another example of the utility of quinine-resistance, several interesting studies have used quinine-resistance to examine how long alcohol drinking must occur to develop compulsion-like motivation for alcohol. We find that Wistar rats need at least 3 months of intermittent-access alcohol (IAA, 24hr/day 3d/week) to develop CLAD (83, 108, 109), although the alcohol intake is the same on 1.5 and 3 months (96, 108). In contrast, Wistar rats exposed to continuous-alcohol access (CAA) schedule for the same number of months do not develop CLAD (108). Interestingly, a subset Lister Hooded rats also require 3 or more months of IAA to develop CLAD (110, 111), with no CLAD development with CAA (110). Further, genetically-selected, alcohol-preferring Marchigian Sardinian P-rats develop quinine-resistance faster than Wistars and Listar Hooded rats, developing CLAD after 1-2 months of IAA but not CAA (112). However, a recent study found that male and female Indiana alcohol-preferring P-rats develop CLAD, with quite high levels of quinine, within 1 week of alcohol drinking (104). Interestingly, P-rat alcohol intake is significantly reduced by quinine the 1st day of consumption (104), suggesting that only brief drinking history is needed for this line of alcohol-preferring rat to develop compulsion for alcohol. However, while Indiana P-rats tolerate 400 mg/L quinine in alcohol after 1 week of drinking, it takes 3–5 weeks of drinking for males to tolerate even higher quinine (500 mg/L), which females won't tolerate. In addition, this rapid development of CLAD in Indiana P-rats (104) is more similar to what is seen with C57 mice, a commonly used line in alcohol studies because of its intake greater than binge level, where we (113) and others (91, 114) find that C57 mice develop CLAD within a few days of alcohol-only drinking. However, it is important to note that these high-drinking P-rat and C57 still have some ability to choose in their CLAD behavior. For example, given a choice between non-adulterated alcohol solution and quinine-adulterated alcohol, Indiana P-rats choose the alcohol-only, suggesting that they still sense the quinine and find this concentration of quinine aversive (115, 116) [also seen in outbred rats (117)]. Similarly, C57 mice will choose alcohol-only over quinine-alcohol after 2 weeks of drinking (called “inflexible”), although by 8 weeks C57 mice are “indifferent” since they drink as much quinine-alcohol as alcohol-only, even though each are freely available (114). See also Pelloux et al. (118) for AIC and quicker onset of cocaine compulsion (88). For some of these studies (including ours), the ability to repeatedly test quinine-resistance across time in the same individual (in a randomized manner) allows critical within-subject comparisons.

## 3. Sex differences in compulsion-like alcohol drinking

Problem drinking and AUD in women has risen dramatically in recent years (2, 28–30). In addition, studies show that women can develop problems related to alcohol misuse sooner and at lower drinking amounts than men, which could involve multiple reasons including social and cultural factors as well as biological contributors, such differences in alcohol pharmacokinetics, levels of sex hormones, and effects of alcohol in the brain (31, 33, 37). However, while clinical studies are extremely valuable for understanding the human condition, it is challenging to disentangle biological and social factors in humans, and thus animal tests have potential to provide valuable insights.

Rodent work often finds that females drink more alcohol than males (65), and there have been some mixed findings regarding sex differences in CLAD [reviewed in Radke et al. (65)]. When drinking under two-bottle choice, female and male C57 mice have similar quinine-resistance across quinine doses (103), which we also observe in rats (69). One important caveat is that understanding aversion-resistance requires that changes in drinking with adverse consequences is normalized to the level of alcohol-only drinking within each individual rodent. With such comparisons, it is thus particularly interesting that, under operant conditions (lever pressing to get alcohol), female C57 mice continue responding for alcohol adulterated with higher concentrations of quinine than males (119). Also, a recent study in the genetically-selected, alcohol-preferring cHAP mouse line found that, when alcohol was paired with 0.25 mA footshock, both female and male mice continue responding for alcohol; however, when the shock increased to 0.35 mA only females keep responding (95). On the other hand, in the genetically-selected, alcohol-preferring Indiana P-rat, females and males overall tolerate high levels of quinine, with males tolerating the higher doses more than females (104). Furthermore, several factors could contribute to lack of sex differences under “simpler” (bottle drinking) vs. more complex (or perhaps less ethological) behaviors such as lever pressing. One interesting possibility is that females focus more on specific, high-value aspects of a task, and less on contextual factors, as recently shown for alcohol (120). In this regard, our recent work with anxiety-like behavior (121), along with other studies [e.g., Olvera-Hernandez and Fernandez-Guasti (122) and Shepherd et al. (123)], suggest that females have a particular focus on more life-relevant aspects of a situation. For example, when a food-restricted rat is in a large arena with food under a bright light, female anxiety-like responding is significantly greater for actual contact with food, with no sex differences in anxiety-like responding a few inches away (in the light but not yet contacting food) (121). It is important to note that, although females can show greater quinine- and footshock-resistance for alcohol, there are no sex differences in (highly reduced) consumption of quinine in water, or in more basic sensitivity to shock (65, 69, 95, 119, 122).

In a recent study from our lab (69), we set out to test the hypothesis that females and males might have different internal action strategies when drinking alcohol. Thus, we analyzed the microstructure of licking in female and male rats and found that female and male licking patterns were overall similar in many aspects, except where females had significantly and substantially (~40%) longer bouts than males. There were both important similarities and differences in sex differences in underlying strategies for obtaining alcohol. More generally, females drank more than males under alcohol-only, moderate-challenge and higher-challenge conditions. However, females had similar total licking as males, suggesting an overall difference in licking efficiency (higher volume per lick in females). Importantly, as noted above, females had significantly longer bouts for AOD and AQ10 compared to males, although this was lost under higher challenge. Interestingly, more persistent responding (longer bouts) is often accompanied by faster licking [see Darevsky et al. (67) and Darevsky and Hopf (68)], and males that licked faster did have longer bouts. However, in strong contrast, females could lick slower or faster and still had significantly longer responding. While unexpected, we have taken these differences to indicate that females can have greater persistence-like responding than males, which does not reflect greater vigor *per se*, and with different strategies under lower vs. higher challenge.

Taken together, these studies suggest that females can have greater levels of consequence-resistant responding for alcohol, although under some (perhaps simpler) conditions there are no sex differences in consequence-resistant responding for alcohol. Finally, these studies suggest that females can use quite different strategies for licking than males, which varies depending on the level of challenge.

## 4. New insights about the neuropharmacology of quinine-adulterated alcohol intake?

In seeking the neural mechanisms that drive compulsion, and also to shed light on novel treatment options for AUD, the Hopf Lab has focused on how CLAD (and other forms of alcohol drinking), might be regulated by orexin-1-receptors (Ox1Rs) [see Radke et al. (65) and Kwok et al. (124)], AMPA-type glutamate receptors (AMPARs) [see Radke et al. (65), Kwok et al. (124) and Hopf and Mangieri (125)], NMDA-type glutamate receptors [NMDARs, see Hopf (26), Wegner et al. (94) and Seif et al. (109)], and α1 and β adrenergic receptors (126, 127). As most of our findings with Ox1Rs, AMPARs, and NMDARs have been previously reviewed elsewhere, we will focus mainly on our newest findings with noradrenergic signaling (with some contrasts to previous results).

The noradrenergic system regulates arousal, stress responses and alcohol addiction (128, 129). Indeed, several human trials have provided compelling evidence for the efficacy of adrenergic receptors to reduce alcohol craving and drives in heavy drinkers. The α1 adrenergic receptor antagonist prazosin is effective against AUD primarily in those with higher alcohol craving and anxiety during withdrawal (130). In addition, prazosin, and a related compound doxazosin, are more effective against alcohol and stress in those with higher blood pressure (131–133), although others have not seen this (134, 135). Prazosin also modulates alcohol drives and negative affect, stress-cue and alcohol-cue activation, and some aspects of autonomic regulation related to alcohol (132, 136–141).

We find that systemic administration of prazosin at the doses of 1.5 and 0.75 mg/kg decreases AOD and CLAD in male rats (127). Also, prazosin (0.3 μg) directly into AIC, an important regulator of the alcohol compulsion in rodents and humans [detailed above, reviewed in Centanni et al. (66)], also significantly decreases both AOD and CLAD without affecting the saccharin intake (127). Interestingly, inhibition of the projection from AIC to the locus coeruleus area in the brainstem, a major source of noradrenaline for the brain, significantly and strongly reduces CLAD, but has no effect on AOD or saccharin intake (127). This is similar to what we observed when inhibiting AIC projections to the nucleus accumbens, which reduces CLAD but not AOD or sweet fluid intake (83). Since AIC projections are linked specifically to CLAD (and not AOD), it was unexpected for prazosin injected within the AIC to inhibit AOD as well as CLAD. Further studies found that global inhibition of AIC with GABA receptor agonists also strongly reduce both AOD and CLAD (but not saccharin intake), indicating that an AIC signaling pathway other than the ones examined thus far (to nucleus accumbens, to locus coeruleus area) is critical for AOD (127). However, while mPFC can contribute to CLAD (83, 109, 115, 142), we found that injection of prazosin into mPFC did not change CLAD or AUD (126).

Although our findings with prazosin do not show specificity to CLAD or AOD (127), we previously demonstrated that lower doses of the Ox1R blocker SB-334867 decreases CLAD in male and female mice (124, 143), while we (124) and others (144) find that higher doses of inhibitor are needed to reduce AOD. Ox1R agents are thus being considered for human addiction treatment [discussed in Giannotti et al. (145)]. In addition, in heavy human drinkers, the NMDAR blocker memantine impacts craving but not simple alcohol intake (146), part of a set of findings, suggesting that a non-canonical NMDAR is linked in rodents and humans to more pathological aspects of alcohol addiction [discussed in Hopf (26)]. As such, we examined systemic administration of D-serine, a canonical positive modulator of NMDARs at the glycine site, with potential for moderate NMDAR regulation. D-serine (300 mg/kg) decreases CLAD but not AOD (although higher D-serine reduces both CLAD and AOD) [(109); see also Wegner et al. (94)]. Surprisingly, we found that injection of D-serine into the nucleus accumbens suppresses CLAD but not AOD, and the canonical NMDAR inhibitor AP5 shows the same behaviorally selective effects, suggesting that D-serine is inhibiting NMDARs to reduce CLAD, and D-serine is known to inhibit non-canonical NMDARs that are linked to human problem drinking (26, 94, 147). Taken together, these findings show that the specificity of a given possible pharmacotherapy to CLAD or AOD will depend on the compound tested as well as the dose used. Interestingly, α1 AR modulation with prazosin impacts both CLAD and AOD, but does not reduce sweet fluid intake. Thus, α1 AR modulating drugs have the potential to be valuable therapeutics and likely produce different impacts on behavior depending on an individual's motivational state (including compulsion-like drinking). Said another way, these drugs have the potential to demonstrate more robustly the presence of sex and individual differences in motivation to drink.

When searching for new therapeutics for drinking problems, it is valuable if a compound is already FDA approved, since it could be more readily repurposed to address alcohol treatment. Prazosin and D-serine (and related modulators) are FDA approved. Interestingly, the so-called beta blockers (for example, the FDA-approved non-selective β adrenergic receptor inhibitor propranolol) have received much less recent attention for alcohol (128, 129). Early studies found efficacy of propranolol against mood disruptions during alcohol withdrawal (128, 148–151), while propranolol has been understudied in recent times [(128, 129, 152, 153), but see Mahabir et al. (154)].

We recently showed (126) that systemic administration of propranolol in male rats decreased CLAD or AOD in a dose dependent manner. While the low dose (2.5 mg/kg) had no effect, the middle dose (5 mg/kg) impacted CLAD more than AOD, and the higher dose (10 mg/kg) reduced both AOD and CLAD. In addition, the β1 adrenergic receptors antagonist betaxolol (BTX) affected CLAD at a lower dose (2.5 mg/kg) while the β2 adrenergic receptors antagonist ICI 118,551 had no effect, suggesting that propranolol is more selectively reducing CLAD than AOD through β1 adrenergic receptors. Interestingly, and different from prazosin, the administration of propranolol into the AIC or medial prefrontal cortex (1–10 μg) had no effects on AOD or CLAD, although there was a trend on the decreasing of CLAD when BTX was injected into AIC. Thus, brain regions where β adrenergic receptors modulators regulate CLAD are worthy of further study.

To better understand how adrenergic receptors promote similar or different aspects of alcohol drinking, one strategy we have used is to test the impact of a given drug on AOD and CLAD within the same animal. For example, one might imagine that some rats have alcohol drinking that is more driven overall by α1 adrenergic receptors, where one would predict that prazosin would cause a large drop in consumption level in both AOD and CLAD. Other rats, with lower α1 adrenergic receptor mediated drive for alcohol, would have little prazosin effects for either drinking condition. However, what we found was very different. Indeed, the prazosin reduction in AOD was not correlated with the prazosin reduction in CLAD, and this was observed for prazosin systemically or within the AIC (127). This is part of the reason (described above) that we speculate there are separable pathways within the AIC for CLAD (which we had expected) and AOD (which was unexpected). Interestingly, we found a similar pattern for β adrenergic receptors modulation (10 mg/kg propranolol), where propranolol reduction of AOD was not correlated with propranolol reduction of CLAD (126). This suggests that two different noradrenergic receptors could both be acting on AOD through different cellular mechanisms than their effects on CLAD. In an interesting convergence, a recent human study (155) used intravenous self-administration in heavy human drinkers to compare two drinking conditions, (1) free choice alcohol drinking, and (2) working for alcohol, where one has to respond more and more across the session to get alcohol (progressive ratio). Remarkably, this study found no relationship between the level of free alcohol and level of working for alcohol across subjects. This is quite similar to the putative disconnection in underlying mechanisms between AOD and CLAD. Thus, it is interesting that people working for alcohol report craving and not enjoying alcohol, while people with free alcohol report enjoying and not craving (and even though total alcohol intake was comparable). Also, more working for alcohol relates to greater family risk of AUD and also some disinhibition measures. While not measures of compulsion, such findings provide some support for the idea of distinct mechanisms for pathological intake and for easier, alcohol-only intake in heavy drinking humans.

In addition to comparing drug effects in AOD vs. CLAD, we are also interested in understanding whether the ability of a particular drug to reduce alcohol drinking is related to the basal level of intake. In other words, it is possible that heavier drinkers recruit different brain mechanisms than more moderate drinkers. Strikingly, we find that the lowest dose of prazosin (0.75 mg/kg) reduces CLAD more in rats with higher basal intake (127), which we also observed with the middle dose of propranolol (5 mg/kg) (126). Thus, α1 and β adrenergic receptor blockers may be more effective at reducing alcohol consumption in higher drinkers, and, in many ways, this is a highly important population to target, since alcohol binging humans account for most of the costs of AUD (see Introduction). Interestingly, we found a similar pattern, with greater reduction in drinking in individuals with higher basal intake, for both the Ox1R blocker (mediated through the nucleus accumbens) (156), and for a novel method to modulate NMDARs (combining D-serine with the FDA-approved sodium benzoate to reduce the breakdown of D-serine) (94). Finally, across our adrenergic receptor studies, we have observed some evidence of U-shaped effects, which are known to occur with noradrenergic signaling [see Valentino and Van Bockstaele (157); detailed in De Oliveira Sergio et al. (126)].

Finally, and importantly, our findings also showed that the combination of sub-therapeutic doses of prazosin and propranolol decreased CLAD and AOD (126); importantly, combined low-dose prazosin and propranolol reduced drinking more in individuals with higher basal alcohol intake. We had shown before that the combination of lower doses D-serine and sodium benzoate decreases CLAD, with no effect on AOD (94). The combination of low dose of these compounds is an interesting strategy since it can decrease the chances of undesirable side effects of higher doses of each drug when given alone [as detailed in De Oliveira Sergio et al. (126)].

## 5. Conclusion

Compulsive drives for alcohol drinking remain a major obstacle for AUD treatment. Animal models of consequence-resistant responding for alcohol can be very useful for understanding the neurobiology related to alcohol consumption as well as to help identify new pharmacological agents for AUD. The two adverse stimuli most used in rodents are quinine-adulteration and footshock-resistance, and we have outlined several advantages of the quinine-adulteration model. Using quinine-adulteration, we find that compounds such Ox1R blockers (at lower doses), NMDARs modulators, and the α1 and β adrenergic receptor antagonists decrease CLAD in rodents. We address our more recent studies where the α1 adrenergic receptor antagonist prazosin also decreases AOD, while a moderate dose of the β adrenergic receptor antagonist propranolol preferentially affected CLAD. We also highlight novel pharmacotherapy strategies which combine lower doses of FDA-approved compounds to target alcohol intake. With the quite limited treatment options available for AUD (17, 18), the significant and critical unmet need for novel therapies (especially FDA-approved drugs that could be quickly repurposed), and the findings outlined above, we have thus demonstrated the need for new clinical therapeutic interventions and highlighted the importance of animal models of compulsion for AUD.

## Author contributions

All authors listed have made a substantial, direct, and intellectual contribution to the work and approved it for publication.

## Footnotes

^1^ https://pubs.niaaa.nih.gov/publications/surveillance101/figures13.htm#f11
